# Analysis of Risk Factors for Multiantibiotic-Resistant Infections Among Surgical Patients at a Children's Hospital

**DOI:** 10.1089/mdr.2018.0279

**Published:** 2019-03-08

**Authors:** Lixin Sun, Suzhe Liu, Jingming Wang, Liqun Wang

**Affiliations:** Management of Hospital Infection Control, Children's Hospital of Hebei Province Affiliated to Hebei Medical University, Shijiazhuang, China.

**Keywords:** multiantibiotic-resistant infections, pediatrics, risk factors, multivariate logistic regression

## Abstract

***Background:*** To identify the potential risk factors for multiantibiotic-resistant infections and provide sufficient evidence for multiantibiotic resistance prevention and control.

***Materials and Methods:*** We conducted a retrospective study of all patients in pediatric orthopedics, pediatric heart surgery, and pediatric general surgery at a level 3, grade A children's hospital from January to December 2016. The clinical laboratory information monitoring system and the medical record system were used to collect patient information regarding age, surgery type, preoperative length of stay, admission season, incision type, preoperative infection, intraoperative blood loss, postoperative use of invasive equipment, duration of catheter drainage, and timepoint of intraoperative prophylactic antibiotics administration. We used logistic univariate and multivariate regression analysis to analyze the potential risk factors for multiantibiotic-resistant infections among pediatric surgical patients. SPSS 21.0 and Excel software packages were used for the statistical analysis.

***Results:*** In total, 2,973 patients met the inclusion criteria: 1,247 patients in pediatric orthopedics, 1,089 patients in pediatric heart surgery, and 637 patients in pediatric general surgery. At the end of the study, 113 patients were multiantibiotic-resistant infection cases; the rate of multiantibiotic-resistant infections was 3.80%, and the detection rate was 84.79%. Multivariate analysis indicated that the multiantibiotic-resistant infection cases were influenced by age, department, admission season, incision type, preoperative infection, and duration of catheter drainage.

***Conclusions:*** Age, department, admission season, incision type, preoperative infection, and duration of catheter drainage may provide possible evidence for prevention and control strategies of multiantibiotic-resistant infections.

## Introduction

Evidence suggests the prevalence of multiantibiotic-resistant bacteria that are difficult to treat with first-line antibiotics has become a growing health care concern and is increasing worldwide,^1–3^ which can occur among children and also enables the selection and spread of clones that carry antibiotic-resistance genes.^4^ In recent decades, several resistant strains have emerged, including strains resistant to aminopenicillins (ampicillin and amoxicillin), chloramphenicols (chloramphenicol and florfenicol), tetracyclines (oxytetracycline and doxycycline), and sulfamethoxazole–trimethoprim.^5^

Data from the Study for Monitoring Antimicrobial Resistance Trends surveillance program show that the global incidence of extended-spectrum beta-lactamase (ESBL)-producing isolates of *Escherichia coli* increased from 9.2% in 2002 to 21.2% in 2010.^6^ Moreover, they found the ESBL-positive rates differed by age group (17.7% in adults vs. 11.4% in children) and by geographic region, with significantly higher rates in Asia/Pacific and significantly lower rates in North America (28.0% vs. 9.1%), specifically.^7^

At present, numerous single-institution studies have emphasized specific types of multiantibiotic-resistant pathogens such as *methicillin-resistant Staphylococcus aureus* (MRSA), *inducible clindamycin resistance*,^8–10^ ESBL,^11^ and *Pseudomonas aeruginosa*,^9^ and so on, that are partly attributable to surgical site infections (SSIs).^11,12^ These infections are the most common complication after surgery and account for >20% of all health care-associated infections in surgical patients.^13^ And there are known host factors (e.g., advanced age, obesity, and diabetes) and procedural factors (e.g., wound class, duration of procedures, and surgical technique) associated with increased risk of SSIs.^14–17^

Much worse, both multiantibiotic-resistant infections and SSIs can result in an increase in mortality and hospital stays primarily in immunosuppressed individuals such as children,^18^ and the rate of these infections in hospitals in developing countries is much higher than that of developed countries because of limited resources.^19^ Furthermore, they also have a major economic impact on health care systems because of the increased costs arising from protracted hospital stays, the greater severity of these infections, and the resulting increase in resource utilization.^20,21^ All the hospital and antimicrobial therapy costs increase when compared with patients with infections because of antimicrobial-susceptible pathogens.^22,23^ Therefore, it is essential to perform studies targeting the risk factors to decrease multiantibiotic-resistant infections.

In this article, we report the findings of a retrospective case–control study that aims to describe the incidence of multiantibiotic-resistant infections among surgical patients, to determine the distribution of common pathogens, and to explore potential risk factors with the goal of reducing multiantibiotic-resistant infections among children in Hebei Province, China.

## Materials and Methods

### Study population

We performed a retrospective case–control study of patients who underwent surgery in the departments of pediatric orthopedics, pediatric heart surgery, and pediatric general surgery at a level 3, grade A children's hospital from January to December 2016. All patients hospitalized for >48 hr were eligible. To prevent the overestimation of multiantibiotic-resistant infections/engraftment, we considered the same multiantibiotic-resistant bacterial strain found more than once in different specimen samples from the same patient as duplication.

### Microbiology surveillance data

All the microbiological tests were conducted by the three departments' laboratories, and all three laboratories shared common methods. The results of antimicrobial susceptibility testing were included in The Star of Clinical Laboratory Information Monitoring System attached to the clinical real-time network at this hospital.

### Surveillance data collection

We collected the surveillance data by using the medical record system, the hospital's acquired infection surveillance system, target surveillance, and reports from clinical medical professionals. We then summarized the data, removed duplication, and organized the cases that were eligible.

### Risk factors for multiantibiotic-resistant infections

We defined cases of multiantibiotic-resistant organisms (MDROs) based on the Technical Guideline for the Prevention and Control of Multidrug-resistant Infections Acquired in Hospital (Trial).^24^ The definition of multiantibiotic-resistant infections excluding colonization or contamination was adopted from the first two in addition to one or more of the rest criteria: (i) the isolation of MDROs, (ii) the presence of compatible clinical signs or symptoms of infections, (iii) the effective therapeutic results of usage of antibiotic, and (iv) the patients with potential or consensual high risks of multiantibiotic-resistant infections.

We identified various potential risk factors for multiantibiotic-resistant infections, including age (≤1 year or >1 year), departments, surgery type (emergency or scheduled), nature of surgery (minimally invasive or conventional), preoperative length of stay, admission season, incision type, duration of operation, preoperative infection, intraoperative blood loss, postoperative use of invasive equipment, duration of catheter drainage, and timepoint of intraoperative prophylactic antibiotics administration.

### Statistical analysis

Proportions were used to summarize binary or categorical data as appropriate. The rate of multiantibiotic-resistant infections was calculated as the number of infections per 100 procedures. A univariate logistic regression was performed to assess the association of each of the potential risk factors with the multiantibiotic-resistant infections. Variables with *p* < 0.20 were considered candidate risk factors to be included in a multivariate logistic regression model. The multivariate logistic regression model was constructed. Variables were eligible for removal at *p* ≥ 0.05.

All the data were analyzed with SPSS (IBM Corp., Armonk, NY) and Microsoft Excel software 2007 (Microsoft Corporation, Redmond, WA).

### Ethics

The study protocol was approved by the Ethics Committee of Children's Hospital of Hebei Province Affiliated to Hebei Medical University (Project No. 2016039).

## Results

### Patient population

In the population analyzed, 2,973 patients met the selection criteria in 2016; 113 cases had multiantibiotic-resistant infections. The rate of multiantibiotic-resistant infections was 3.80% (113/2,973), and the detection rate was 84.33% (113/134).

### Pathogens

In this study, we discovered five pathogens for multiantibiotic-resistant infections. The incidence of ESBL was 1.98%, making it the most common pathogen; it was distributed mainly in the departments of pediatric heart surgery and pediatric general surgery. The second-most common pathogen was MRSA with an incidence of 1.51%, which was distributed mainly in the department of pediatric orthopedics. The results demonstrated a significant difference in the incidence of multiantibiotic-resistant pathogens (*p* < 0.05) (Table 1), and the pathogens' distribution in the same department also showed a markedly significant difference (*p* < 0.05) (Table 2).

**Table 1.** Pathogens Causing Multiantibiotic-Resistant Infections of Surgical Patients in 2016

*Pathogen*	N	*Incidence (%)*	*χ*^2^	p
MRSA	45	1.51	93.98	<0.001
ESBL	59	1.98		
CRE	5	0.17		
CRAB	3	0.10		
MDR/PDR-PA	1	0.03		

CRE, carbapenem-resistant *Enterobacteriaceae*; CRAB, carbapenem-resistant *Acinetobacter baumannii*; MDR/PDR-PA, multidrug-resistant/pandrug-resistant *Pseudomonas aeruginosa*; ESBL, extended-spectrum beta-lactamase; MRSA, methicillin-resistant *Staphylococcus aureus*.

**Table 2.** Distribution of Pathogens Causing Multiantibiotic-Resistant Infections of Surgical Patients in 2016

*Departments*	*MRSA*		*ESBL*		*CRE*		*CRAB*		*MDR/PDR-PA*		*χ*^2^	p
	N	*%*	n	*%*	n	*%*	n	*%*	n	*%*		
Orthopedics												
Yes	35	2.81	2	0.16	0	0	0	0	0	0	129.85	<0.001
No	1,212	97.19	1,245	99.84	1,247	100	1,247	100	1,247	100		
Heart surgery												
Yes	0	0	34	3.12	5	0.46	3	0.28	1	0.09	96.25	<0.001
No	1,089	100	1,055	96.88	1,084	99.54	1,086	99.72	1,088	99.91		
General surgery												
Yes	10	1.57	23	3.61	0	0	0	0	0	0	62.96	<0.001
No	627	98.43	614	96.39	637	100	637	100	637	100		
χ^2^	2931.66		2719.20		2952.58		2960.72		2968.90			
*p*	<0.001		<0.001		<0.001		<0.001		<0.001			

### Risk factors for multiantibiotic-resistant infections based on univariate logistic regression analysis

The univariate analysis identified 13 variables as potential risk factors for multiantibiotic-resistant infections. The results showed that the risk factors significantly associated with multiantibiotic-resistant infections were age, department, admission season, incision type, duration of operation, nature of surgery, surgery type, preoperative infection, postoperative use of invasive equipment, duration of catheter drainage, and timepoint of intraoperative prophylactic antibiotics administration (*p* < 0.20). In contrast, risk factors that were determined not to be significantly associated with multiantibiotic-resistant infections were preoperative length of hospital stay and the volume of intraoperative blood loss (*p* > 0.05) (Table 3).

**Table 3.** Analysis of the Potential Risk Factors for Multiantibiotic-Resistant Infections (*N* = 2,973)

*Risk factors*	*Infections (*n* = 113)*		*Odds ratio*	p
	*Yes*	*No*		
Age			0.45	<0.001
≤1 year	60	965		
>1 year	53	1,895		
Departments				
Orthopedics	37	1,210		0.075
Heart surgery	43	1,046	1.276	0.284
General surgery	33	604	1.738	0.023
Surgery type			0.222	<0.001
Emergency	48	403		
Scheduled	65	2457		
Preoperative length of stay			1.199	0.396
≤3 days	81	2,151		
>3 days	32	709		
Admission season				
Spring	25	1,009		0.024
Summer	26	562	1.867	0.028
Autumn	19	483	1.588	0.135
Winter	43	806	2.153	0.003
Incision type				
Clean	44	1,509		<0.001
Clean contaminative	30	1,162	0.885	0.612
Contaminative	19	141	4.621	<0.001
Dirty	20	48	14.29	<0.001
Duration of operation			1.949	0.01
<3 hr	94	2,595		
≥3 hr	19	265		
Preoperative infection			0.146	<0.001
Yes	76	661		
No	37	2,199		
Intraoperative blood loss			1.104	0.849
<50 mL	109	2,768		
≥50 mL	4	92		
Nature of surgery			0.155	0.009
Minimal	111	2,563		
Conventional	2	297		
Postoperative invasive equipment			0.528	0.001
Yes	68	1,269		
No	45	1,591		
Duration of catheter drainage				
No	22	1,735		<0.001
<3 days	28	760	2.906	<0.001
≥3 days	63	365	13.612	<0.001
Timepoint for prophylactic antibiotics				
No	8	1,337		<0.001
0.5–1 hr postoperatively	57	1,121	8.498	<0.001
Other	48	402	19.955	<0.001

### Risk factors for multiantibiotic-resistant infections using multivariate logistic regression analysis

Based on the results of the univariate analysis, 11 risk factors identified in univariate analysis were further analyzed by multivariate logistic regression analysis. This analysis identified six parameters as independent markers for the occurrence of multiantibiotic-resistant infections: age, departments, admission season, incision type, preoperative infection, and duration of catheter drainage >3 days (Table 4).

**Table 4.** Analysis of Risk Factors for Multiantibiotic-Resistant Infections Using Multivariate Logistic Regression Model

*Risk factors*	*B*	*SE*	*Wals*	*df*	*Sig.*	*Exp (B)*
Age	−0.522	0.233	5.002	1	0.025	0.593
Departments						
Orthopedics			13.973	2	0.001	
Heart surgery	2.778	0.962	8.33	1	0.004	16.08
General surgery	1.759	0.492	12.79	1	0	5.809
Surgery type	−0.606	0.341	3.164	1	0.075	0.545
Admission season						
Spring			15.616	3	0.001	
Summer	1.026	0.354	8.395	1	0.004	2.79
Autumn	0.624	0.372	2.807	1	0.094	1.865
Winter	1.078	0.286	14.215	1	0	2.939
Incision type						
Clean			22.912	3	0	
Clean contaminative	0.651	0.73	0.795	1	0.373	1.918
Contaminative	1.846	0.803	5.286	1	0.021	6.333
Dirty	2.796	0.831	11.308	1	0.001	16.38
Duration of operation	0.377	0.309	1.484	1	0.223	1.457
Preoperative infection	1.037	0.261	15.758	1	0	2.817
Nature of surgery	−1.284	0.753	2.903	1	0.088	0.277
Postoperative invasive equipment	0.524	0.458	1.309	1	0.253	1.69
Duration of catheter drainage						
No			21.725	2	0	
<3 days	0.361	0.442	0.666	1	0.415	1.435
≥3 days	1.312	0.349	14.128	1	0	3.712
Timepoint for prophylactic antibiotics						
No			4.116	2	0.128	
0.5–1 hr postoperatively	0.281	0.48	0.342	1	0.559	1.324
Other	0.809	0.487	2.764	1	0.096	2.246

df, degrees of freedom; SE, standard error.

## Discussion

In recent decades, many studies have reported that the increasing incidence of serious infections acquired in health care settings are now associated with antimicrobial drug resistance, which now constitutes a serious threat to global public health.^25^ In China, various multiantibiotic-resistant infections seem to be on an uninterrupted incline,^26–28^ and recent data suggest that multiantibiotic-resistant infections are also increasing in children.^29^ These MDROs that commonly infect humans have multiple resistance mechanisms and mainly include the following microorganisms: *Enterococcus faecium, S. aureus*, *Klebsiella pneumoniae*, *Acinetobacter baumannii*, *P. aeruginosa*, and *Enterobacter* spp.^30^ It has been reported that overuse of the same type of antimicrobial agents, including prophylactic use to prevent SSIs or infections associated with urogenital diseases, may have caused the emergence of resistant strains.^31^

In this study, we revealed that the incidence of ESBL was the highest, followed by MRSA, carbapenem-resistant *Enterobacteriaceae* (CRE), carbapenem-resistant *A. baumannii* (CRAB), and multidrug-resistant (MDR)/pandrug-resistant *P. aeruginosa* (PDR-PA) in descending order. ESBL cases accounted for 52.21% of all the multiantibiotic-resistant infections cases, which showed the prevalence of the existing multiantibiotic-resistant pathogens and the distribution in our hospital. These findings were consistent with the studies of Hoban *et al.* and Hsueh *et al.*, who showed the high incidence of ESBL, too.^32,33^ Moreover, ESBL-producing *E. coli* was also found to be common in community settings in the epidemiological research of Doi *et al*.^29,34^

These results make it clear that the number of children with ESBL infections is increasing both in hospital settings and in the community because of selection pressure from broad-spectrum antibiotic use. Pediatricians in both the hospital and the community must be aware of the resistance trends of ESBL in their patient populations and determine the appropriate therapy and dosing for MDROs to treat these challenging infections.^29^

After hospital discharge, a patient may carry MDROs, whether they are commensal, such as *Staphylococcus epidermidis*, or truly pathogenic, such as MRSA. According to other investigations, MRSA is a common pathogen associated with soft tissue infections,^35^ when the skin's surface is disrupted and the bacteria have an easy mode of entry into the body, an infection may develop. Therefore, the most common diagnoses associated with MRSA infections in the specialty of orthopedics include cellulitis, abscess, postoperative SSI, and infections resulting from a surgically implanted device, which also explains the results of our study to a certain extent.^36^ In addition, it is always important to take essential steps to prevent the evitable infections caused by MRSA mentioned previously after being hospitalized at our hospital.

The increasing spread of multiantibiotic-resistant infections in the hospital setting is promoted by several factors in our study. In the multivariate logistic regression analysis, we considered younger age and preoperative infection as independent risk factors for multiantibiotic-resistant infections in our study. It is well-known that because of the lack of exposure to microbes and microbial products early in life, the immunity of neonates remains weak, and the risk of various infections may increase with early-life immune stimulation, which is in line with our findings.^37^ Furthermore, when a patient has a preoperative infection, we hypothesize that he or she is more likely to develop multiantibiotic-resistant infections because of the direct infectivity of their own pathogens through self-contact transmission or blood-stream transmission. In addition, when a preoperative infection causes low immunity at admission, colonized pathogen carriers more frequently develop multiantibiotic-resistant infections, a finding that is in agreement with previous reports.^38^

Besides, MDR bacteria are more frequently associated with nosocomial infections,^39^ and infections caused by MDR pathogens have been associated with an increase in mortality and morbidity rates, along with an increase in hospital stay and the need for chronic care.^40–42^ In modern times, many of our current medical practices and treatments directly contribute to overall increases in infections; such practices include the increased use of prophylactic antimicrobials (which increases the risk of resistance), more invasive procedures, increased use of vascular access and other devices, and more immunosuppression for oncologic and rheumatologic conditions.^43–45^ In our investigation, there were similar results. When surgery was performed in the department of heart surgery—a higher level type of surgery accompanied by greater technical difficulties, more complicated invasive procedures, and greater danger when compared with surgery in the departments of orthopedics and general surgery—the risk of multiantibiotic-resistant infections escalates.

We also discovered that with an increased duration of catheter drainage, the risk of development of multiantibiotic-resistant infections becomes higher. In addition, the surgical incision classification serves as an independent factor for multiantibiotic-resistant infections, and when the incision is more contaminative, the risk of multiantibiotic-resistant infections increases gradually. These findings remind those of us in the hospital settings that measures to prevent and control the increase of multiantibiotic-resistant infections and the dissemination of resistance genes are crucial.^39^

As mentioned previously, several effective infection control strategies can assist in reducing multiantibiotic-resistant infections, and one of these strategies is reducing the duration of catheter drainage after an appropriate and timely assessment by a pediatrician. Moreover, Sadsad *et al*. reported that hospitals should apply a wide range of infection control policies to reduce the burden of nosocomial infections. These policies include contact precautions, creating cohorts by grouping staff with particular patients or allocating staff or patients to designated areas, surveillance for multiantibiotic-resistant pathogens, decolonization treatment to reduce the carriage of MRSA in patients, restricted or strategic use of antimicrobials,^46^ and postoperative wound management to prevent fecal contamination and avoid the development of multiantibiotic-resistant infections in pediatric infections.^47^ In China, the implementation the Management Rules of Antimicrobial Usage by the Chinese Ministry of Health on July 1, 2011 provides a unique opportunity to observe the effects of reduced antimicrobial prophylaxis use for all surgeries.

According to studies, long-term decreases in air quality are associated with statistically and clinically significant negative effects on children's health, such as lung function damage^48,49^ and increased arterial blood pressure and hypertension.^50^ Hebei Province has emerged as one of the most polluted areas in China because of its geographical location and urban development in recent years.^51^ Indeed, air quality is closely related to not only the quantity but also the time and location of pollutant emissions and the meteorological conditions.^52^ In Hebei, haze is most severe during the period from winter to spring; when the air quality is poorest, it carries the high risk of multiantibiotic-resistant infections in patients undergoing surgeries at our hospital.

The overuse and misuse of antimicrobials in human medicine is a major cause of multiantibiotic resistance.^53^ However, it has been reported that the use of antimicrobial prophylaxis could control the rate of SSIs and has an inverse effect on the potential nosocomial infection rate.^54^ In this study, although an association was found between the timepoint of prophylactic antibiotics use and multiantibiotic-resistant infections, this association unfortunately failed to reach statistical significance (*p* > 0.05). Hence, we advocate for reductions in the use of antibiotic use, even for prophylaxis, as much as possible, and recommend conducting more experiments and studies to address this controversy.

Here are some possible limitations for this article including the collection of retrospective data from a small sample that may have biased our results; the missing data of the types of multiantibiotic-resistant infections restricting further study and this was a single-center study, which may not be applied to other patient populations or the pediatrics in general hospital completely.

In summary, this study suggests the inclusion of risk factors for multiantibiotic-resistant infections, namely younger age, undergoing major surgery, admitted in severe haze periods, with long catheter drainage duration, and with preoperative infections and intraoperative incision contamination. Moreover, targeted strategies to decrease the incidence of preventable multiantibiotic-resistant infections are needed to further improve the quality and safety of surgery at our hospital and similar hospitals elsewhere.
